# Similar Perceptions on Continuous Glucose Monitor Use amongst Youth with Type 1 and Type 2 Diabetes

**DOI:** 10.1155/2023/1979635

**Published:** 2023-06-19

**Authors:** Alexander Phu, Tyger Lin, Jacquelyn A. Manfredo, Elizabeth A. Brown, Risa M. Wolf

**Affiliations:** ^1^Department of Medicine, Kansas City University, Kansas City, USA; ^2^Department of Pediatrics, Division of Endocrinology, Johns Hopkins University School of Medicine, Baltimore, USA

## Abstract

**Methods:**

Youth with T1D and T2D (currently on insulin therapy) without current CGM participated in a prospective CGM study and were given a series of questionnaires when starting CGM intervention. BenCGM and BurCGM questionnaires assessed the participant's perspectives on continuous glucose monitor use, while DDS surveys assessed participants' QoL associated with diabetes. Survey results were compared between T1D and T2D groups, and multivariable analysis was used to assess differences in perceptions of continuous glucose monitor use in youth with diabetes.

**Results:**

Participants with T1D (*n* = 26, 65.4% male, 42.3% non-Hispanic black, median age 14.2 years, median HbA1c 10.3%) and T2D (*n* = 41, 39% male, 80.5% non-Hispanic black, median age 16.2 years, median HbA1c 10.3%) scored similarly on the BenCGM, BurCGM, and DDS surveys. In a pooled analysis of both T1D and T2D, there was no difference in survey results by race/ethnicity, but female youth had an increased odd of diabetes-related distress, specifically regimen-related distress.

**Conclusions:**

Youth with T1D and T2D on insulin therapy report similar perspectives on continuous glucose monitor use and QoL measures. Insulin use in both T1D and T2D may carry a similar burden of management, and CGM may help improve quality of life. Trial registration: This trial is registered with NCT04721145, NCT04721158.

## 1. Introduction

Over the last two decades, there has been an increasing incidence and prevalence of youth onset type 1 (T1D) and type 2 diabetes (T2D) [1, 2]. Youth with T1D require insulin therapy and frequent blood glucose monitoring to maintain recommended glycemic trends [3]. Youth with T2D also often require insulin therapy and glucose monitoring with a potentially similar burden of management [4]. Continuous glucose monitoring (CGM) improves glycemic trends and reduces complications of diabetes in youth with T1D, while also improving quality of life (QoL) [5, 6]. Although there have been studies on the perceptions of CGM in pediatric patients with T1D, the perceptions of CGM in pediatric T2D patients are limited [7, 8]. We compared baseline survey results from youth with diabetes participating in a prospective CGM trial in order to assess the perception of continuous glucose monitor use and QoL measures amongst youth with T1D and T2D.

## 2. Methods

This study was approved by the institutional review board of the Johns Hopkins University School of Medicine. As previously described [9], this was a preregistered prospective CGM trial where questionnaires were given to participants with T1D and T2D who were starting a trial continuous glucose monitor, and the results of questionnaires at their baseline visits were compared. Eligible participants were ages 5 to 21 and had T1D without prior use with CGM or had not used CGM in more than 9 months, or were youth with T2D on insulin therapy with no prior use of CGM. Participants were enrolled from January 20, 2021, through February 21, 2022. Of those approached, 67/79 (84.8%) agreed to participate. Participants were given questionnaires including the Hypoglycemia Confidence Scale (HCS), Glucose Monitoring Satisfaction Survey (GMSS), perceived benefits of CGM scale (BenCGM), perceived barriers of CGM scale (BurCGM), Pediatric Quality of Life Diabetes Module, version 3.2 (PedsQL), and Diabetes Distress Scale (DDS) [10]. BenCGM, BurCGM, and PedsQL surveys were previously validated in youth with diabetes and measured items such as distress, efficacy, and attitudes towards continuous glucose monitor use [10–12]. GMSS, HCS, and DDS, although commonly used in studies in youth with T1D and T2D, have been validated in the adult diabetes population [13–15]. Participants with T2D were given a T2D-specific version of the GMSS and DDS questionnaires. PedsQL Child and Teen versions were given based on age.

For all participants, the following demographic and clinic data were collected: age, sex, race/ethnicity, insurance type, parental education, household income, duration of diabetes, diagnosis age, insulin administration type, previous CGM, and need for smartphone for CGM. For participants with T2D, the use of insulin, metformin, or liraglutide was also recorded.

The primary outcome of this study was to compare perspectives on CGM and QoL measures between participants with T1D or T2D.

### 2.1. Statistical Analysis

Analysis was performed using SAS version 9.4. Categorical variables are described by frequency and percentage of the total. Continuous variables have been evaluated for normality using the Shapiro–Wilk test. Normally distributed variables are described by mean and standard deviation, while non-normal variables are described by median and interquartile range. Chi-square or Fisher's exact tests were used to evaluate the differences in categorical descriptive characteristics. Continuous descriptive variables and crude association between type of diabetes and survey results were evaluated using two sample *T*-tests for variables with a normal distribution, or Wilcoxon rank sum tests, for non-normally distributed variables. Multivariable linear regression was used to model surveys. Residuals were checked for normality. All DDS surveys were modeled using logistic regression analysis. Models were fit to predict severe distress, score >3 on the DDS scale. *P* value <0.05 is considered statistically significant.

## 3. Results

### 3.1. Clinical Characteristics

As shown in Table 1, 67 youth (*n* = 26 T1D, *n* = 41 T2D) were included in this study. Youth with T1D were 65.4% male, 42.3% Non-Hispanic (NH) Black, median age 14.2 years (IQR 12.5–16.7, range 8.5–19.8), with median HbA1c of 10.3% (IQR 8.6–12.5). Youth with T2D were 39% male, 80.5% NH black, median age 16.2 years (IQR 15-17.5, range 11.2–18.6), with median HbA1c of 10.3% (IQR 7.3–12.5). For participants with T2D, nearly all were already utilizing insulin (97.6%) at the time of the visit, and one participant was prescribed insulin at the visit when CGM was started. As shown in Table 1, there were differences in age, sex, race, duration of diabetes, age at diagnosis, and household income between the T1D and T2D cohorts.

### 3.2. Comparison of Survey Results

Comparison of survey scores between the T1D and T2D participants (Table 2) showed similar scoring on the BenCGM, BurCGM, GMSS, total DDS score, DDS subscores of emotional burden, physician-related distress, regimen-related distress, interpersonal distress, and PedsQL questionnaires (*P* > 0.05).

### 3.3. Multivariable Analysis

In a pooled analysis of T1D and T2D survey results, multivariable regression models adjusting for age, sex, race, diabetes type, diabetes duration, and insurance, female youth reported lower average scores on the PedsQL questionnaire (OR −8.07; Cl, −16.00 to −0.14; *P*=0.046), and specifically, lower average diabetes symptom QL subscores (OR −12.38; Cl, −20.65 to −4.12; *P*=0.004) compared to male youth. Female youth also had 11.7 times the odds of overall severe distress, with DDS score >3 (OR 11.7, CI, 1.05 to 130.17 *P*=0.045). The overall severe DDS is driven primarily by higher odds of regimen-related distress (OR 14.4; CI, 1.49 to 139.39; *P*=0.021). For both T1D and T2D groups, each year of increased duration of diabetes was associated with 1.44 times the odds of severe distress, DDS > 3 (OR 1.44; CI, 1.01 to 2.07; *P*=0.047), driven primarily by 1.77 times the odds of severe interpersonal distress for each year of duration (OR 1.77; CI, 1.13 to 2.78; *P*=0.014). Additionally, youth who were on private insurance had a lower average adjusted openness score in the GMSS questionnaire compared to those on public insurance (OR −0.48; Cl, −0.87 to −0.08; *P*=0.018) and higher odds of severe emotional distress (OR 4.12; Cl, 1.11 to 15.39; *P*=0.035). In a pooled analysis of both T1D and T2D, there were no significant unadjusted or adjusted differences in survey results by race and ethnicity.

## 4. Discussion

Youth with T1D and T2D had similar scores on questionnaires related to CGM and QoL, indicating that perceptions of CGM and QoL measures may be similar in these cohorts. While all youth with T1D are managed with insulin therapy, in this population, all participants with T2D were also utilizing insulin (and 35/41 (85%) of youth with T2D were utilizing basal/bolus insulin), suggesting that youth with T1D and T2D (on insulin) may experience a similar burden of management [16]. One recent study showed that CGM may improve QoL for adolescents with T2D on a stable medication regimen [8]. The similarities in PedsQL questionnaire scores amongst the two groups show that for youth with T2D that use insulin, the impact of diabetes on QoL may be comparable to youth with T1D, attributable to a similar burden of management and the experience of similar lifestyle interruptions. BenCGM and BurCGM scores were similar amongst youth with T1D and T2D, suggesting similar perceptions of the benefits of CGM in diabetes management and glucose monitoring amongst all youth with diabetes.

Despite known disparities in glycemic trends, health-related outcomes, and use of diabetes technology between white and nonwhite youth with diabetes [17], we did not find a difference in survey results when stratifying by race. Specifically, in this group of participants who all accepted to try a sample continuous glucose monitor, there was no difference in perception of the burdens or benefits of CGM by race, highlighting the importance of the provider recommendation for patients to consider CGM [17]. Further, each 1-year increase in diabetes duration was associated with 1.4 times the odds of severe diabetes-related distress, which may be related to the daily burden of diabetes management [18].

Similar to other studies, we found that female youth report a lower QoL associated with T1D [19]. It was found that female youth living with chronic illness like T1D reported elevated stress related to their illness compared to male youth with the same chronic condition [20]. In our cohort, female youth scored an average of 12.4 points lower on the symptom-related PedsQL questionnaire and 8.1 points lower on the overall PedsQL questionnaire compared to male youth and increased odds of diabetes-related distress, specifically, regimen-related distress. While we did not find a gender difference in the results of the BenCGM and BurCGM surveys, prior studies show that female youth experience a greater burden when utilizing CGM compared to males, citing concerns about body image and self-consciousness [21, 22]. Female participants had worse scores on the PedsQL and DDS questionnaires which may indicate a need to incorporate additional clinical or psychosocial support to promote QoL in this population [23].

Although this was a prospectively conducted study with a diverse patient population, this study has limitations. The sample size was small, only collected from two clinic sites, and consisted of patients who were mostly CGM naïve. This study cohort only included youth who agreed to start CGM by design and thus may not be generalizable to a larger population of pediatric patients with diabetes. As a survey study without open-ended responses, there may have been a potential lack of depth and an inability to expand upon patient perceptions that were not directly addressed through survey questions. Of the youth with T1D, 19.2% had prior insulin pump use, and past experience with diabetes technology may have informed participants' survey results, although this factor was not directly assessed. Future studies comparing perceptions of CGM and QoL measures after CGM may contribute to our understanding of how CGM impacts psychosocial factors, and how psychosocial factors impact sustained CGM amongst patients with T1D and T2D.

## Figures and Tables

**Table 1:** Characteristics of participants when survey completed.

Variable	Type 1 diabetes ( *N* = 26)	Type 2 diabetes (*N* = 41)	*p* value
	*N* (%)	*N* (%)	
Age (years), median (IQR)^*∗*^	14.2 (12.5–16.7)	16.2 (15–17.5)	0.006
Sex, male	17 (65.4%)	16 (39%)	0.04
Race/ethnicity			0.02
NH white	11 (42.3%)	4 (9.8%)	
NH black	11 (42.3%)	33 (80.5%)	
Hispanic	3 (11.5%)	4 (9.8%)	
Mixed race	1 (3.8%)		
Public insurance	17 (65.4%)	31 (75.6%)	0.36
Parent education (T1: *n* = 24, T2: *n* = 40)			0.62
≤High school diploma	16 (66.7%)	29 (72.5%)	
>High school diploma	8 (33.3%)	11 (27.5%)	
Household income <$50,000 (T1: *n* = 21, T2: *n* = 32)	9 (42.9%)	26 (81.3%)	0.01
HbA1c %, median (IQR)	10.3 (8.6–12.5)	10.3 (7.3–12.5)	0.32
Duration of DM (years), median (IQR)	4.6 (2.4–7.7)	0.8 (0.4–2.7)	<0.0001
Age at diagnosis (years), mean (SD)	8.4 (3.8)	14.2 (2)	<0.0001
Insulin pump use	5 (19.2%)		
Previous continuous glucose monitor use	11 (42.3%)		
On insulin^*∗∗*^		40 (97.6%)	
On metformin		33 (80.5%)	
On liraglutide		2 (4.9%)	

Not all participants reported parental education and household income. ^*∗*^Ben CGM and BurCGM surveys were validated in youth ages 12–19 years. PedsQL survey was validated in youth ages 2–25 years. GMSS, DDS, and HCS surveys are validated in adults with T1D. and T2D. ^*∗∗*^One participant with Type 2 diabetes was started on insulin at the visit when CGM was placed.

**Table 2:** Results of variables of completed surveys.

Questionnaire description	Type 1 diabetes							Type 2 diabetes						
Variable	*N*	Mean (std dev)	Median	Minimum	Maximum	Lower quartile	Upper quartile	*N*	Mean (std dev)	Median	Minimum	Maximum	Lower quartile	Upper quartile
Perceived benefits of CGM scale (BenCGM)	26	4 (0.6)	4.1	3.1	5	3.6	4.3	41	4.1 (0.7)	4.1	2	5	3.7	4.7
Perceived Burdens of CGM Scale (BurCGM)	26	2 (0.6)	2	1	3.1	1.5	2.3	41	1.9 (0.7)	2	1	3.9	1.4	2.4
The glucose monitoring satisfaction survey (GMSS)-openness scale	26	3.5 (0.7)	3.4	2.3	5	3	4	41	3.4 (0.7)	3.3	1.8	5	3	3.8
The glucose monitoring satisfaction survey (GMSS)-emotional burden scale	26	2.1 (0.6)	2.1	1	3.8	1.5	2.3	41	2.2 (0.8)	2	1	5	1.5	2.5
The glucose monitoring satisfaction survey (GMSS)-behavioral burden scale	26	2 (0.7)	2	1	3.3	1.8	2.3	41	2 (0.8)	2	1	4.3	1.3	2.5
Diabetes distress scale DDS^*∗*^	26	1.7 (0.8)	1.4	1	4.2	1.1	2.1	37	2 (1)	1.9	1	5	1.2	2.5
Diabetes distress scale DDS^*∗*^-emotional burden subscale	26	2.2 (1.2)	2	1	5.2	1.2	3.4	37	2.5 (1.4)	2	1	5.2	1.2	3.6
Diabetes distress scale DDS^*∗*^-physician related distress	26	1.3 (0.6)	1	1	3.5	1	1	37	1.5 (0.9)	1	1	5	1	2.3
Diabetes distress scale DDS^*∗*^-regimen related distress	26	1.8 (1.1)	1.5	1	5	1	2	37	2.2 (1.2)	1.6	1	5	1.2	2.8
Diabetes distress scale DDS^*∗*^-interpersonal distress	26	1.4 (0.8)	1	1	4	1	1.3	37	1.6 (1.1)	1	1	5	1	1.7
QL diabetes	25	71.6 (14.5)	73.4	34.4	95.3	63.3	82	40	71.1 (15.9)	75	23.4	97.7	59.8	80.1
QL diabetes management subscore	25	79.3 (15.6)	85.3	41.2	100	70.6	91.2	40	72.9 (18.9)	76.5	20.6	100	62.5	86
QL diabetes symptom subscore	25	62.9 (15.9)	66.7	26.7	100	51.7	71.7	40	69 (17.4)	70.8	26.7	100	56.7	80.8

^*∗*^Data is not normally distributed.

## Data Availability

The data supporting the current study are available from the corresponding author upon request.
